# The effects of whole mushrooms during inflammation

**DOI:** 10.1186/1471-2172-10-12

**Published:** 2009-02-20

**Authors:** Sanhong Yu, Veronika Weaver, Keith Martin, Margherita T Cantorna

**Affiliations:** 1 Center for Immunology and Infectious Disease, Department of Veterinary and Biomedical Science, The Pennsylvania State University, University Park, PA, USA; 2 Department of Nutrition, Arizona State University, Mesa, AZ, USA

## Abstract

**Background:**

Consumption of edible mushrooms has been suggested to improve health. A number of isolated mushroom constituents have been shown to modulate immunity. Five commonly consumed edible mushrooms were tested to determine whether whole mushrooms stimulate the immune system *in vitro *and *in vivo*.

**Results:**

The white button (WB) extracts readily stimulated macrophage production of TNF-α. The crimini, maitake, oyster and shiitake extracts also stimulated TNF-α production in macrophage but the levels were lower than from WB stimulation. Primary cultures of murine macrophage and ovalbumin (OVA) specific T cells showed that whole mushroom extracts alone had no effect on cytokine production but co-stimulation with either lipopolysacharide or OVA (respectively) induced TNF-α, IFN-γ, and IL-1β while decreasing IL-10. Feeding mice diets that contained 2% WB mushrooms for 4 weeks had no effect on the *ex vivo *immune responsiveness or associated toxicity (changes in weight or pathology of liver, kidney and gastrointestinal tract). Dextran sodium sulfate (DSS) stimulation of mice that were fed 1% WB mushrooms were protected from DSS induced weight loss. In addition, 2% WB feeding protected the mice from transient DSS induced colonic injury. The TNF-α response in the colon and serum of the DSS challenged and 2% WB fed mice was higher than controls.

**Conclusion:**

The data support a model whereby edible mushrooms regulate immunity *in vitro*. The *in vivo *effects of edible mushrooms required a challenge with DSS to detect small changes in TNF-α and transient protection from colonic injury. There are modest effects of *in vivo *consumption of edible mushrooms on induced inflammatory responses. The result is not surprising since it would certainly be harmful to strongly induce or suppress immune function following ingestion of a commonly consumed food.

## Background

There are thousands of different mushroom species and about 700 species have been reported to have significant pharmacological properties [1,2]. Medicinal mushrooms have a long history of use in traditional Oriental therapies. Hot-water-soluble fractions of medicinal mushrooms have been used as medicine in the Far East [3]. In addition, mushroom extracts have begun to be sold as dietary supplements with a world-wide market value of around 5–6 billion US dollars per year [4].

Isolated mushroom constituents have been shown to have beneficial effects on experimental cancer. Efforts have been made to isolate and purify the active and protective components of mushrooms. Many of these compounds are large polysaccharides or β-(1→6)-branched β-(1→3)-linked glucans. The β-glucans have been shown to inhibit tumor growth *in vitro *and *in vivo*. The β-glucans lentinan from *Lentinus edodes*, schizophyllan (sonifilan) from *Schizophyllum commune*, grifolan from *Grifola frondosa*, and extracts from *Sclerotinia sclerotiorum *all have anti-tumor activity. Intratumor injection of an acid-treated fraction of *Agaricus blazei *inhibited tumor growth of that tumor as well as other tumors at remote sites [5]. An extract from the *Phellinus rimosus *mushroom extended the life span of mice by 96% following injection of tumor cells in an experimental Dalton's lymphoma ascites model [6]. Injection of a methanol crude extract from *Lepista inversa *also increased life span by 50% in a lymphocyte leukemia model [7]. Extracts of multiple varieties of mushrooms have been shown to be protective in experimental cancer models; presumably because in part they boost anti-tumor immunity. Whether these same benefits of mushrooms can be derived from whole mushrooms instead of the isolated components is not known.

There is considerable information and research on identifying the biologically active components of medicinal mushrooms and using them as therapies and immune system modulators. What is less clear is whether the active components of edible mushrooms are present in adequate amounts to show benefits when consuming whole mushrooms or using extracts from whole mushrooms *in vitro*. Furthermore, there are concerns about the toxicologic side effects of whole mushrooms especially the *Agaricus *species that might counter-indicate recommending eating mushrooms. The aims of the present study were 1) to determine what the effects of whole mushroom extracts were on the cytokine profile of macrophage and T cell cultures *in vitro*, 2) to determine whether feeding mice diets that contained 1–2% whole mushrooms resulted in measurable changes in immunity, and 3) to determine whether 2% whole mushrooms diets led to pathologic findings in tissues likely to be affected (liver, kidney, gastrointestinal tract).

## Results

### Induction of tumor necrosis factor (TNF)-α in RAW 264.7 cells by extracts of commonly consumed mushrooms

**Figure 1:**
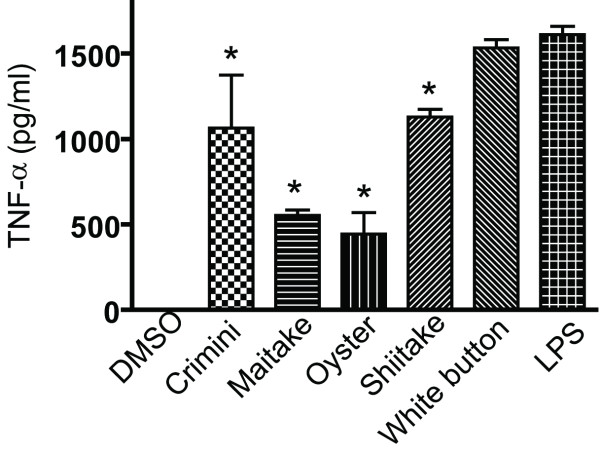
**Production of TNF-α by RAW 264.7 cells**. Mushroom extracts (100 μg/ml), LPS or vehicle (DMSO) controls were added to Raw 264.7 cells and 72 h later TNF-α production was evaluated in triplicate cultures. Data from one representative of three individual experiments are shown. Values are mean ± SD, *p < 0.05 compared to the LPS TNF-α response.

The mouse macrophage cell line (RAW 264.7) was treated with mushroom extract (100 μg/ml) or the vehicle DMSO. Cell viability was high following treatment (90–95%). Lipopolysacharide (LPS) was used as a positive control to induce production of TNF-α from RAW cells. The white button (WB) mushroom extracts readily stimulated TNF-α production from RAW 264.7 cells at levels comparable to the LPS stimulated cells (Fig. 1). The crimini, maitake, oyster and shiitake extracts all stimulated TNF-α production from the RAW 264.7 cells; however, the amount of TNF-α produced was significantly less than stimulation with the WB mushroom extracts (Fig. 1).

### Mushroom regulated cytokine production in bone marrow derived macrophage (BMDM)

**Figure 2:**
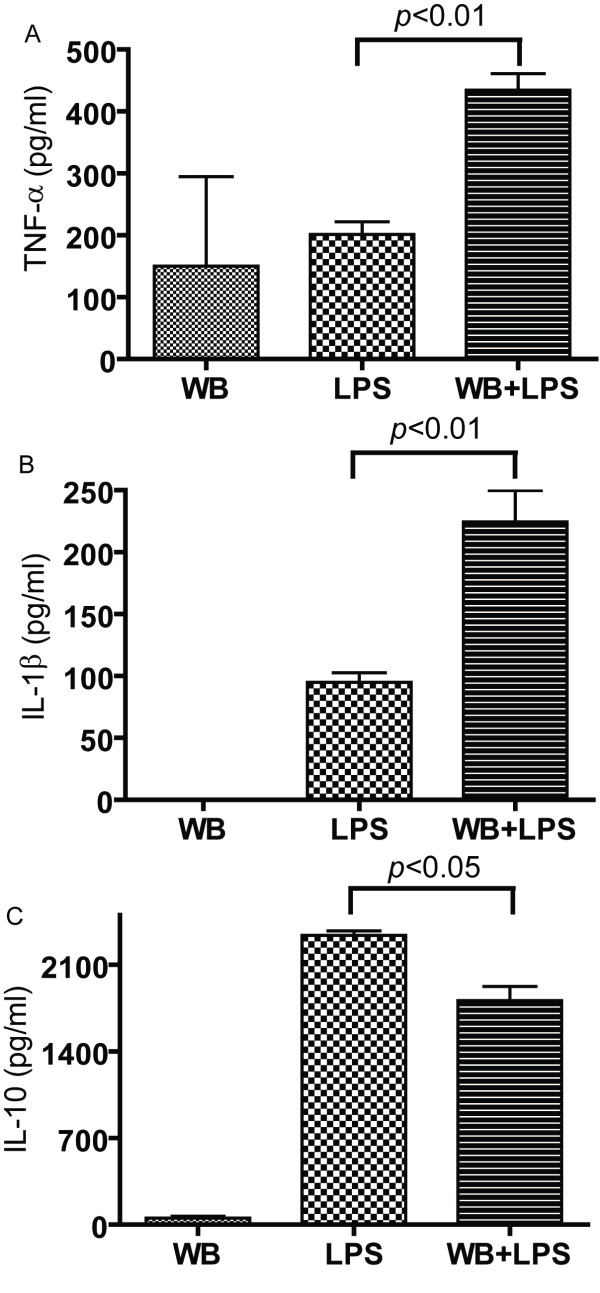
**Cytokine production by primary BMDM**. WB mushroom extracts alone, LPS alone or WB in combination with LPS (WB+LPS) were added to BMDM. Supernatants were harvested 72 h later and (A) TNF-α, (B) IL-1β and (C) IL-10 were detected by ELISA. The data is one representative of four individual experiments. Values are mean ± SD of triplicate cultures. Values for WB+LPS are significantly different from LPS stimulated with the p value as shown.

Follow up experiments used macrophage isolated from the bone marrow (BM) of wildtype (WT) mice. The data from WB mushroom extracts is shown since the highest TNF-α secretion from RAW 264.7 cells was found in the WB stimulated cultures (Fig. 1). The BMDM cells are untransformed mouse macrophage that produce a variety of cytokines. Three cytokines produced by macrophage were picked for analysis: TNF-α, interleukin (IL)-10 and IL-1β (Fig. 2). BMDM cells plus DMSO (control) cultures did not produce TNF-α, IL-10 or IL-1β (data not shown). Like the results from the RAW 264.7 cell line, WB extracts alone stimulated TNF-α production. LPS stimulation also induced TNF-α and the combined effect of WB plus LPS was additive for TNF-α secretion (Fig. 2A). For IL-1β production, WB stimulation alone had no effect, LPS stimulation alone induced IL-1β and WB plus LPS increased the secretion of IL-1β (Fig. 2B). WB stimulation induced a small amount of IL-10, while LPS stimulated a robust IL-10 response and interestingly enough WB addition to the LPS containing cultures inhibited IL-10 production (Fig. 2C). BMDM were also treated with crimini and shiitake extracts and the results were similar to those shown in Fig. 2 for WB mushrooms (data not shown). The data suggest that WB, crimini and shiitake mushrooms had similar effects on cytokine production from BMDM.

### Mushroom induced T cell production of cytokines

**Figure 3:**
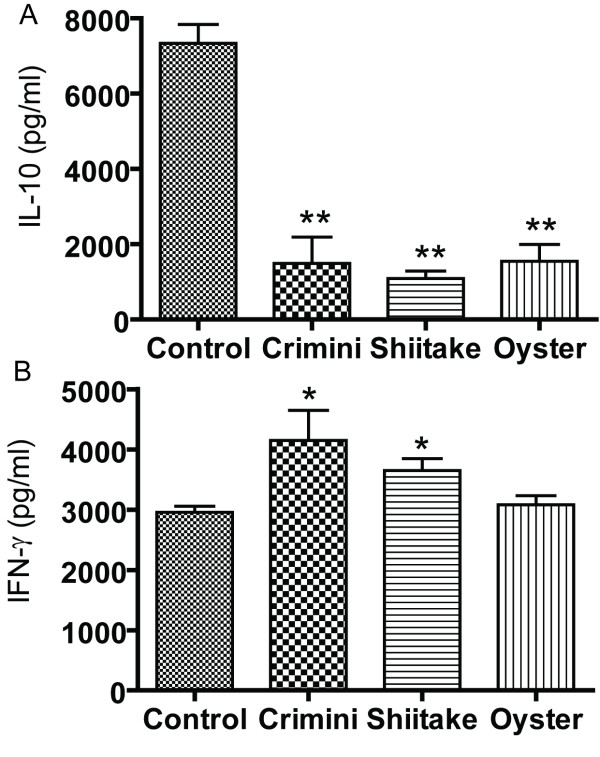
**Mushroom mediated inhibition of IL-10 and enhancement of IFN-γ**. Splenocytes from OT II mice were stimulated with OVA plus WB, Crimini, Shiitake, Oyster and Maitake extracts or Control (OVA plus DMSO). (A) IL-10 and (B) IFN-γ secretion. Representative data from two individual experiments with n = 5–6 mice each. Values are mean ± SEM. Values are significantly different from the control group, *p < 0.05, **p < 0.01.

Crimini, shiitake and oyster mushrooms were used to stimulate antigen specific T cells. T cell receptor transgenic (OT II) mice were used as a source of antigen (ovalbumin, OVA) specific T cells. The lymphocytes from the spleen of OT II mice were isolated and stimulated with OVA plus DMSO (control), or OVA plus mushrooms. In the absence of OVA stimulation the splenocytes did not produce cytokines. OVA stimulation (control) induced IL-10 production that was inhibited by crimini, shitake and oyster mushroom extracts (Fig. 3A). Conversely, interferon (IFN)-γ secretion was induced by crimini, shiitake and oyster mushroom extracts in two experiments and unaffected by mushroom extracts in another (Fig. 3B). IL-4 was undetectable in all of the supernatants tested. The experiment was repeated using OVA stimulation alone (DMSO control) or WB stimulation. IFN-γ secretion was 2058 ± 60 pg/ml in the controls and 2534 ± 154 pg/ml in the WB stimulated cultures while IL-10 was 3500 ± 50 pg/ml in the controls and 1200 ± 135 pg/ml in the WB stimulated cultures (control IL-10 values are significantly higher than WB IL-10 values P < 0.05). The data suggest that in these mixed cell cultures all 4 types of mushrooms either increased IFN-γ or had no effect. In addition, there were no differences between the ability of the 4 types of mushrooms tested for inhibiting IL-10 in T cells.

### Orally ingested mushrooms do not show overt toxicity or cytokine production *ex vivo*

**Figure 4:**
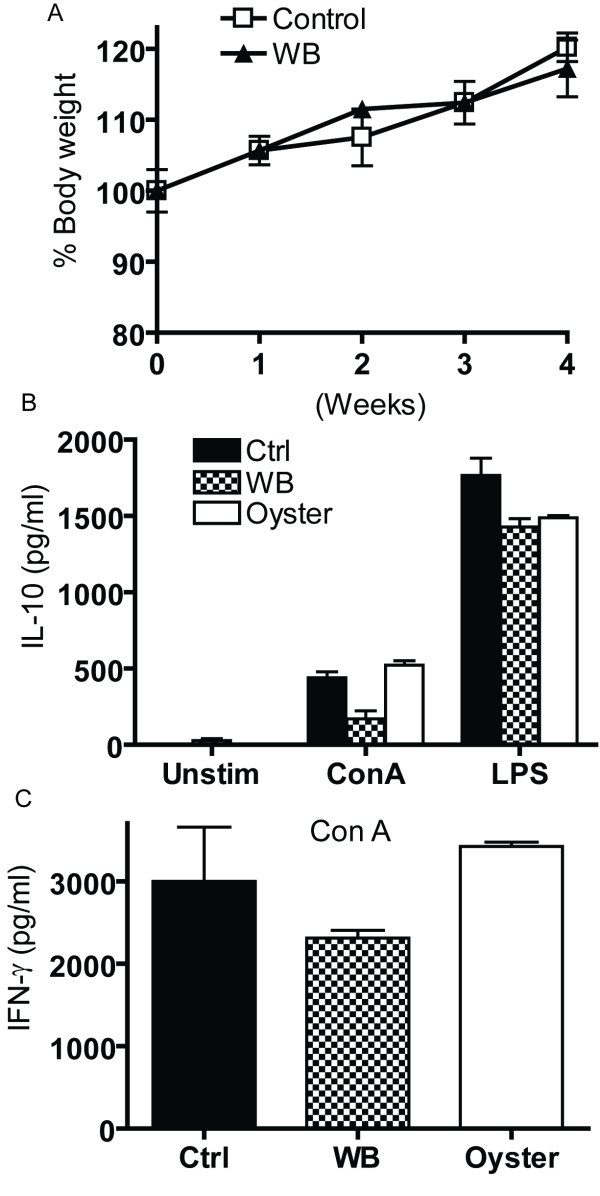
**In vivo effects of feeding mushrooms**. C57BL/6 mice were fed control diet only or control diet that contained 2% WB freeze dried mushrooms. (A) Weights of the WB fed mice gained weight over the 4 weeks of the study. Values are mean ± SEM with 6–10 mice per group. (B) IL-10 and (C) IFN-γ production of Con A and LPS stimulated splenocytes from 1% WB fed, 1% oyster fed and control (Ctrl) fed mice. Values are mean ± SEM with 3–5 mice per group.

The *in vivo *experiments were done primarily with WB mushrooms and one experiment was done using oyster mushrooms. The weight curves show that WB mushroom containing diets (2% by weight) did not negatively affect body weight and instead the mice continued to grow over the 4 weeks of the study (Fig. 4A). Histopathology sections of the stomach, intestine, kidney, and liver showed that feeding WB mushrooms for 4 weeks was not associated with any pathology in these tissues (data not shown). Unchallenged mice had undetectable levels of TNF-α, IL-10 and IL-1β in the colon or blood whether or not they were fed WB mushrooms (data not shown). Splenocytes were removed from the WB and oyster fed mice and cell surface staining showed that there was no change in the percentage of CD4+, CD8+, B cells, and macrophage as a result of including mushrooms in the diet (data not shown). The splenocytes from the WB, oyster and control fed mice were stimulated *in vitro *with the T cell mitogen concanavalin (Con) A or the macrophage/B cell mitogen LPS. Splenocytes did not make IL-10 without further restimulation *in vitro *(Fig. 4B). Con A stimulation induced IL-10 in cultures from all 3 groups of mice but there was no effect on IL-10 production as a result of the mice consuming mushrooms (Fig. 4B). LPS stimulation induced considerably more IL-10 than Con A but still all 3 groups of mice produced IL-10 and there were no significant differences across the groups (Fig. 4B). There was no detectable IL-12 in the Con A or LPS stimulated cultures, low levels of TNF-α secretion and no IFN-γ production in the LPS stimulated splenocytes. Splenocytes did not make IFN-γ without further restimulation *in vitro *(data not shown). Con A stimulated splenocytes from the control (Ctrl), WB and Oyster treated groups produced IFN-γ (Fig. 4C) but there was no effect of WB or oyster mushrooms compared to Ctrl treatment on the *ex vivo *IFN-γ response.

### Dextran sodium sulfate (DSS) treatment and *in vivo *mushroom feeding

**Figure 5:**
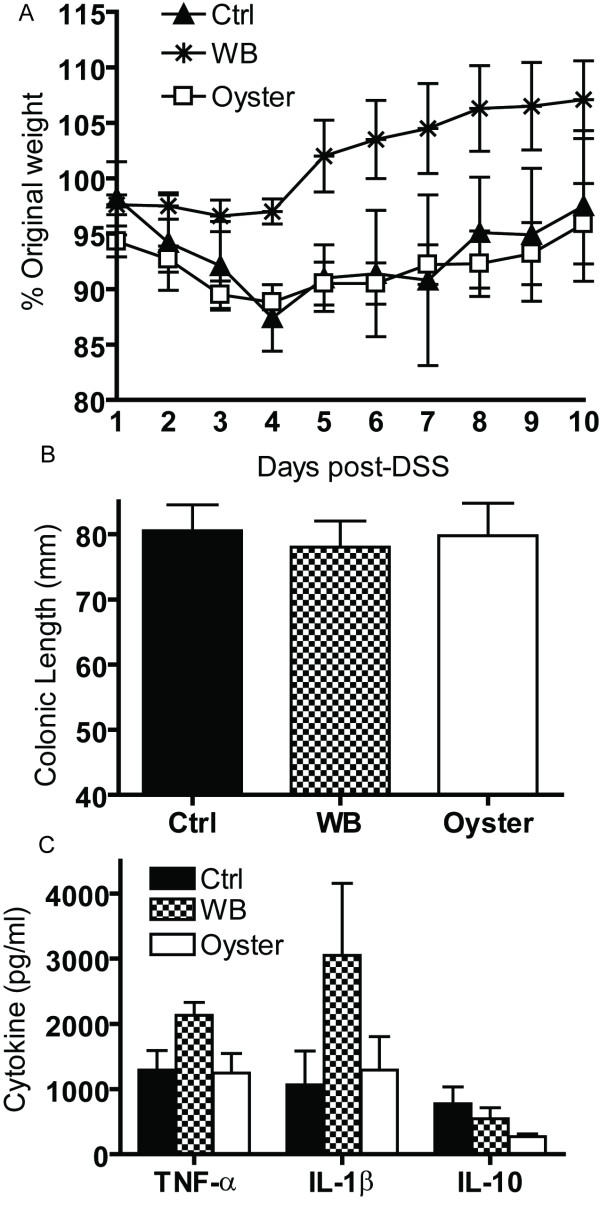
**DSS challenge of 1% WB, 1% oyster and control fed mice**. Control, WB and oyster fed mice that were treated with DSS for 5 d followed by water for 5 d (10 d post-DSS). (A) Percentage of starting weight following DSS treatment, (B) colonic length at d 10 after DSS treatment, and (C) colonic production of TNF-α, IL-1β and IL-10 at d 10 post-DSS. Values are mean ± SEM of 4–6 mice per group.

Based on the data in Fig. 2 showing the maximal effect of WB mushrooms occurred when the BMDM are also stimulated with LPS, mice were challenged *in vivo *with DSS. Like LPS, DSS stimulates the innate immune system. DSS initiates mucosal epithelial cell damage by disrupting barrier function, leading to ulceration, bleeding, and transient shortening of the colon [8,9]. The damage to the gut mucosa begins to repair following removal of the DSS from the water. Consistent with the acute colitis induced by DSS the control fed mice showed a drop in weight after 4 d of DSS treatment and then the mice recovered in weight almost completely by 10 d post-DSS (Fig. 5A). Weight loss in the oyster fed mice was not different from the control mice (Fig. 5A). Conversely, the WB fed mice did not lose as much weight as the other 2 groups (significantly different at d4, d5, and d6 and not different at d10, Fig. 5A). Additional groups of mice were fed control, 1% WB and 1% oyster diets but were not exposed to DSS. All Mice from all three groups that were not exposed to DSS maintained their body weight throughout the experiment (data not shown). There was no bleeding observed in any of the colons upon sacrifice at d10. The colons of WT mice fed control, 1% WB or 1% oyster diets and not exposed to DSS averaged 79 mm in each group (data not shown). Ten d post-DSS treatment the control, WB and oyster fed mice had colons that were not different from one another and averaged 78–79 mm (Fig. 5B). Colonic homogenates showed that all groups produced TNF-α, IL-1β and IL-10 but that the variability was such that there were no significant differences (Fig. 5C). Colonic homogenates from mice that were not treated with DSS produced low levels of TNF-α (700 pg/ml), and IL-10 (400 pg/ml, IL-1β was undetectable) and not different in the control, WB and oyster fed mice (data not shown).

**Figure 6:**
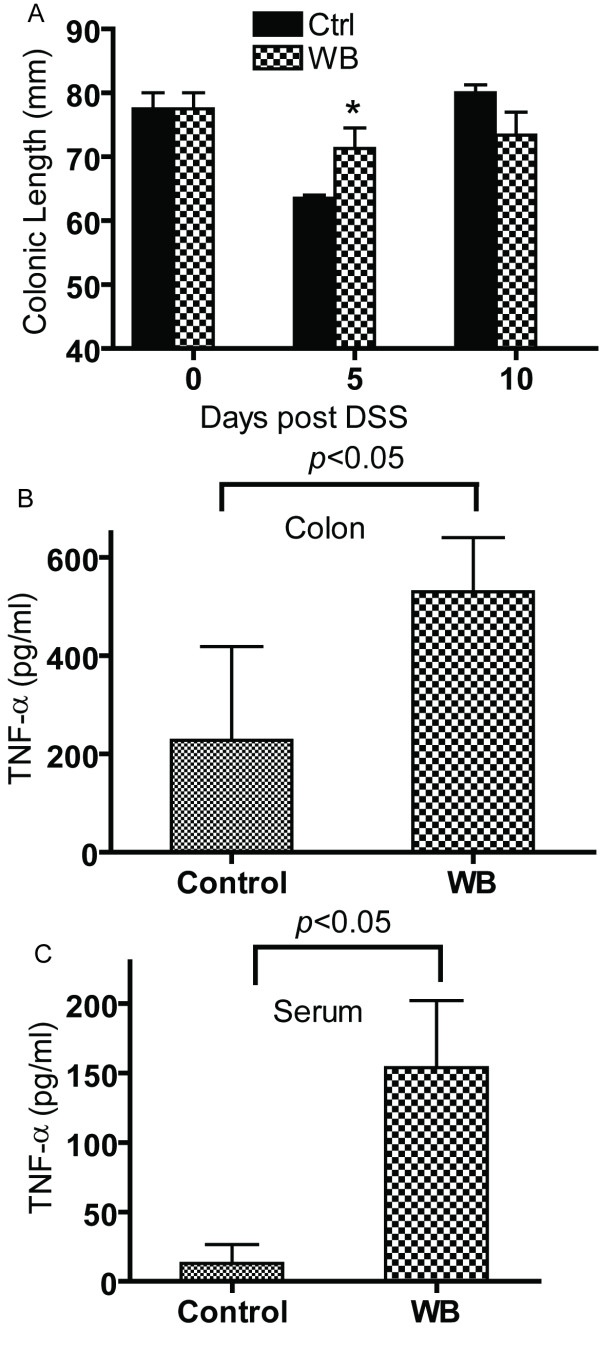
**DSS colitis of 2% WB fed and control fed mice**. Control, and WB fed mice were treated with DSS for 5 d (5 d post-DSS) followed by water for 5 d (10 d post-DSS). (A) Colonic length as a function of DSS treatment, TNF-α production at d 10 post-DSS in the colon (B) and serum (C). Values are mean ± SEM of 4–8 mice per group. Values are significantly different from the control group, *p < 0.05.

The DSS experiment was repeated using 2% WB fed mice and control fed mice. There was no blood in the colons of WB or control fed mice at 5 or 10 d post-DSS (data not shown). The colonic lengths of the control fed mice shortened as expected following 5 d of DSS (from 78 to 64 mm) and the WB fed colons were also shorter following 5 d of DSS (from 78 to 71 mm) however the colons from WB fed mice were significantly longer than control at d5 post-DSS (Fig. 6A). At d 10 the WB and control colons recovered and were not different from one another (Fig. 6A). The amount of TNF-α produced in the colon and blood of WB fed mice was significantly higher than the controls (Fig. 6B and 6C, respectively). Other cytokines including IL-1β, IL-10 and IFN-γ, were not affected by WB feeding (data not shown). Histopathology of the colon showed no effect of WB feeding on symptoms of DSS colitis. WB feeding of WT mice was associated with protection from early weight loss (Fig. 5A), protection from colonic shortening (Fig. 6A) and an increase in TNF-α production in the serum and colon (Fig. 6B).

## Discussion

**Figure 7:**
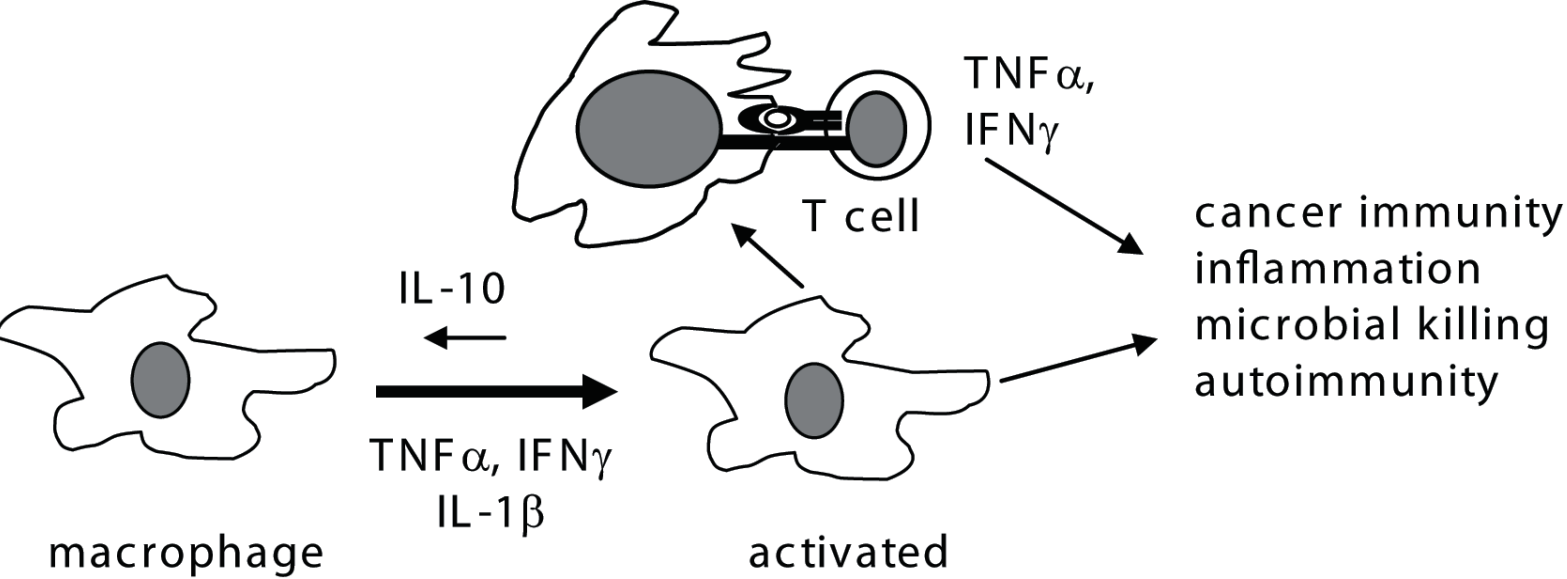
**Model of the effects of whole mushrooms on anti-cancer immunity**. Whole mushrooms contain active components that can induce TNF-α, and IL-1β *in vitro *while inhibiting IL-10 production. Consuming mushrooms in the diet also had an effect on immune function but that effect is evident only when the immune system is challenged. The change in the immune response induced by whole mushrooms is consistent with a potentially important improvement in cancer surveillance and anti-microbial killing while increasing inflammation and perhaps autoimmunity.

The immunomodulatory or antitumor activity associated with mushroom intake has been suggested to be due to a number of isolated fractions from mushrooms including the β-D-glucans, and other polysaccharides [10-13]. The standard approach has been to purify the components and then to test them for efficacy. It is likely that mushrooms possess multiple immunoregulatory components including the selenium, B vitamins and polysaccharides that when ingested together or mixed *in vitro *together would have effects that differed from the isolated components. *In vitro *WB, crimini, shiitaki, and oyster extracts reduced IL-10 production and increased IL-1β, and TNF-α production by macrophage. The activation of the macrophage with the mushroom extracts preferentially stimulated T cell production of TNF-α and IFN-γ and very little IL-10. This pattern of immuno-regulation by mushrooms is consistent with a model whereby whole mushroom consumption would induce a modest but important boost in immune responses that would improve anti-cancer immunity (Fig. 7). In macrophage, the inhibition of IL-10 and activation of IFN-γ, IL-1β, and TNF-α would result in an activated phenotype for the macrophage that would induce production of T cell and macrophage cytokines like IFN-γ and TNF-α that are important in clearance of tumorogenic cells, pro-inflammatory signaling, and killing of infectious organisms (Fig. 7). In RAW 264.7 cells the induction of TNF-α production was highest in LPS and WB stimulated rather than the crimini, maitake, oyster, or shitake stimulated cells. The differences might be a result of differences in the quantity of the immunomodulatory substance or substances and or differences in solubility of mushrooms in the DMSO used to produce the extracts. However, the direction of the changes (increase *versus *decrease) were not different among the mushroom treatments. The lack of differential effects on cytokine secretion by the mushroom extracts suggests that the whole mushrooms must share a common component(s) that act to regulate immune function.

Feeding mice diets that included up to 2% WB mushrooms over 4 weeks had no effect on a number of immune parameters including percentage of T and B cells, Con A and LPS stimulated cytokine production and colonic expression of a panel of cytokines including IL-1β, IL-10, TNF-α and IFN-γ. This is consistent with data by Wu et al. that showed that WB mushroom feeding at much higher doses (2–10%) and for 10 weeks did not affect T cell, B cell, NK cell, and macrophage cell numbers or affect Con A and LPS induced cytokine production [14]. This is not completely unexpected since it would certainly be harmful to have a commonly present dietary component induce or suppress normal immune function.

DSS is normally used to cause transient colonic injury and as a model of acute colitis. WB feeding was protective for some parameters of DSS colitis; early weight loss and colonic shortening. It is unclear by what mechanism the WB feeding would protect from colonic injury. When challenged with DSS the WB mushroom fed animals transiently produced more TNF-α in the colon but showed no other changes in immune function. TNF-α production is negatively associated with colitis symptoms in the DSS model. Therefore the increased local production of TNF-α and decreased colitis injury following WB intake are paradoxical. The improvement in colitis symptoms is unlikely to be related to the increased production of TNF-α but instead might reflect a change in the composition of the bacterial microflora that has been shown to impact disease symptoms in the DSS model [15]. The data show that WB mushrooms are protective against colonic injury and the mechanisms underlying the protective effects would be an area worthy of future investigation.

Agaritine, a natural compound found in the *Agaricus *species of mushrooms, has been implicated as a carcinogen [16-18]. Even though animal studies do not show agaritine to be a carcinogen at physiological doses there is still a possible concern regarding toxicity at high intake levels of mushrooms [16-18]. WB mushrooms are *Agaricus *mushrooms and are the most commonly consumed mushrooms in the US. Toxicity might be expected in tissues that come into contact with the agaritine like the stomach or intestines and those tissues that might break down or excrete absorbed agaratine like the liver or kidney. Based on the normal growth curves of mice fed 2% WB mushrooms for 4 weeks and data by Wu et al. showing that intake as high as 10% WB mushrooms for 10 weeks did not affect body weight we can conclude that there are no gross effects of feeding mice agaritine containing mushrooms [14]. In addition, histopathology sections of the stomach, small intestine, large intestine, liver and kidney from mice fed WB mushrooms showed no changes that might indicate an affect of increased agaritine intakes over the 4 weeks of the study. While it still might be possible that longer term intake of agaritine containing mushrooms might prove toxic, the present data using moderate doses of mushrooms does not support the claim.

Mushrooms contain a significant amount of protein, vitamins and minerals in addition to a large amount of carbohydrate much of which are polysaccharides that are indigestible by humans and therefore are considered dietary fiber [19]. It is possible that differences in the cultivation of mushrooms in different regions and by different growers may affect the composition of an important immunomodulatory factor. One of the limitations of the present study is that the results presented may only reflect results of mushrooms grown in Pennsylvania and more specifically by the manufacturers of those mushrooms in Pennsylvania. Future research should sample mushrooms from different suppliers of the same species for comparison of immunomodulatory functions.

Several major substances have been isolated from mushrooms and shown to be immunomodulatory. What has not been considered is that mushrooms have a number of bacteria, yeasts and molds associated with them. In fact microorganisms are required for initiation of fruit body formation. Normal healthy mushrooms have high bacterial populations associated with them with the numbers ranging from 6.3–7.2 log CFU/g of fresh mushrooms [19]. The bacteria associated with mushrooms are predominately the pseudomonads [19-22]. The pseudomonads have been shown to activate the innate immune response by interactions with the Toll like receptors (TLR), especially TLR-5 [23-25]. It is likely that the microbiota ingested with the mushrooms are triggering the mucosal innate immune response through the TLR. More research is required to determine the relative contributions of bacteria, *versus *nutrient and other components of mushrooms as immune system regulators.

## Conclusion

Whole mushrooms have a number of components that are potentially immuno-modulatory. The *in vitro *data show that whole mushroom extracts regulate macrophage and T cell production of cytokines in a way that is predicted to be beneficial for boosting anti-tumor immunity. *In vivo*, the immuno-regulatory functions of edible mushrooms are harder to detect. Following challenge with DSS there is a transient protection from colonic injury and a modest increase in TNF-α production locally in the colon. Whether the increase is an effect of known immuno-modulatory nutrient components or as a result of bacteria like the psuedomonads that are associated with mushroom cultivation is not known.

## Methods

### Animals

OTII (OVA specific T cell receptor transgenic) C57BL/6 and control C57BL/6 mice were bred and maintained at the Pennsylvania State University (University Park, PA). The mice were fed synthetic diets made in the laboratory as described previously [26-28]. In one experimental design the mice were either control fed or fed diets that included 2% powdered mushrooms for 4 weeks (8–10 mice per group). In a second design mice were fed control, 1% or 2% WB or oyster mushrooms for 1 week prior to, and continuing through the DSS treatment (4–8 mice per group). Controls included additional mice that were fed the same diet but were not treated with DSS (3–4 mice per group). Experimental procedures were approved by the Office of Research Protection, Institutional Animal Care and Use Committee at the Pennsylvania State University.

### Mushroom extracts

Commercially available and commonly consumed mushrooms were used for this study. *Agaricus bisporous *or common name WB, brown *Agaricus bisporous *or common name crimini, *Grifola frondosa *or common name maitake, *Lentnula edodes *or common name shiitake, and *Pleurotus eryngii *common name king oyster mushrooms were obtained from Modern Mushroom Farm, Inc. (Toughkenamon, PA). The whole mushrooms were freeze-dried and ground into a fine powder. For the *in vitro *experiments, 10 mg of mushroom powder was suspended in 1 ml dimethyl sulfoxide (DMSO, Sigma, St. Louis, MO) and sonicated (Branson Ultrasonic Corporation, Danbury, CT) for 8 min at room temperature. The crude unfiltered extracts were used for the experiments below. Mushroom extracts were tested for LPS levels (Limulus Ameobocyte Lysate assay, Cambrex, Walkersville, MD). There was a low level of LPS contamination of the extracts (3 ng/ml in WB and 3.2 pg/ml or less in the other extracts). The final concentration of LPS from the WB extracts added to the cultures was 30 pg/ml LPS. This concentration of LPS was well below the level of LPS required to stimulate cells in vitro. Although commercially produced according to standardized methods, there are likely to be important differences between mushrooms grown in different areas and under different conditions. Thus, freeze-dried mushrooms and extracts will be provided upon request.

### Cell cultures

RAW 264.7 cells were obtained from ATCC (Manassas, VA) and 10^6 ^cells per well were cultured in the presence of 100 μg of mushroom extracts or equal concentrations of DMSO (0.1 μl/ml) alone for 72 h in Dulbecco's modified Eagle's medium (Sigma) supplemented with 10% fetal calf serum (FCS), 100 U/L penicillin, 100 mg/L streptomycin and 4 mmol/L glutamine (Invitrogen, Carlsbad, CA). Viability of the cultures after the 72 h was checked by trypan blue exclusion and was not different between the cells that received mushroom extract, DMSO or just media.

BMDM were obtained as previously described [29]. BMDM from five C57BL/6 mice were pooled and cultured for 7 d at 37°C in the presence of recombinant macrophage colony-stimulating factor (R&D Systems, Minneapolis, MN), in cold DMEM medium containing 25 mmol/L HEPES, 2 mmol/L glutamine, 5 × 10^-5 ^mol/L 2-mercaptoethanol, 100 U/L penicillin and 100 mg/L streptomycin, supplemented with 100 ml/L FCS. More than 90% of the cells had macrophage morphology (large cells with irregular outlines, abundant cytoplasm and oval or indented nucleus) as determined by viewing cytospin preparations and by flow cytometry (F480 and CD11b positive). After 7 d in culture, the BMDM were washed with phosphate-buffered saline (PBS) and cells were cultured (10^6 ^cell/well) in the presence of mushroom extracts (100 μg/ml) alone, LPS (0.5 μg/ml, Sigma) alone, or mushroom extracts plus LPS for 72 h. Cell viability was checked at 72 h (trypan blue exclusion) and found not to be different among the different treatment groups.

Splenocyte suspensions from OT II mice were prepared and placed (10^6 ^cell/well) in complete RPMI 1640 (Sigma) medium-supplemented with 100 ml/L FCS, mmol/L HEPES, 2 mmol/L glutamine, 100 U/L penicillin and 100 mg/L streptomycin (Invitrogen, Gibco). Splenocytes in 24-well culture plates (Becton Dickinson Labware, Franklin Lakes, NJ) were cultured in the presence of crimini, shiitake, or oyster (100 μg/ml) and OVA (1 mg/ml, Sigma) or OVA alone for 72 h. In a separate series of experiments OT II splenocytes were cultured with WB (100 μg/ml) and OVA (1 mg/ml, Sigma) stimulation or OVA alone for 72 h. Cell viability was checked at 72 h (trypan blue exclusion) and found not to be different among the different treatment groups.

Splenocytes suspensions from C67BL/6 mice were prepared and 10^6 ^cells/well were placed in complete RPMI 1640. Splenocytes were cultured with LPS, 0.5 μg/ml), Con A (10 μg/ml) or media for 72 h when supernatants were collected for the detection of cytokines by enzyme-linked immunosorbent assay (ELISA). Cell viability was checked at 72 h (trypan blue exclusion) and found not to be different among the different treatment groups.

### DSS challenge

Mice were administered 3.5% DSS (MW = 40 kDa; ICN Biomedicals, Solon, OH) dissolved in filter-purified and sterilized water ad libitum for 5 d followed by a return to water for the remainder of the experiment (10 d following the start of DSS treatment).

### Colon measurements

The gross colonic blood scoring system previously described by Siegmund *et al*[30] was used. The entire colon from cecum to anus was removed and the length was measured and reported as colonic length as described [8].

The distal colon was weighed and the same amount of tissue was cut open and washed in 1 × PBS containing penicillin (100 U/ml) and streptomycin (100 mg/ml). Tissue was then scrapped in 1 ml PBS using a razor blade. The colon tissue scrapings were centrifuged at 10,000 *g *at 4°C for 10 min. Cytokine concentrations in the supernatant of the colonic homogenate were measured by ELISA.

### Histopathology

Parts of the stomach, intestine, kidney, and liver were saved in 10% formalin and sent to the Pennsylvania State Diagnostic Laboratories (University Park, PA) for staining (hematoxilyn and eosin) and evaluation of the tissue sections. The distal colon were removed from mice treated with DSS and histological analysis was performed blinded for pathology associated with DSS treatment as described [9].

### Cytokine detection

ELISA kits (Pharmingen, San Diego, CA) were used to determine the levels of IFN-γ, IL-10, IL-1β, TNF-α, IL-4 and IL-12 in the supernatants, serum and colonic homogenates. The limits of detection were 125 pg/ml IFN-γ, 31.25 pg/ml IL-10, 31.25 pg/ml IL-1β, 31.25 pg/ml TNF-α, 31 pg/ml IL-4 and 125 pg/ml IL-12 p70.

### Statistical analysis

Statistical analyses were performed using PRISM software (GraphPad Software, San Diego, CA) using ANOVAs and data that were not normally distributed were transformed prior to ANOVA. P values of 0.05 or less were considered statistically significant.

## Abbreviations

BM: bone marrow; BMDM: bone marrow-derived macrophages; Con: Concanavalin; Ctrl: control; DSS: dextran sodium sulfate; ELISA: enzyme-linked immunosorbent assay; FCS: fetal calf serum; IFN: interferon; IL: interleukin; LPS: lipopolysaccharide; PBS: phosphate buffered saline; OVA: ovalbumin; TNF: tumor necrosis factor; WB: white button; WT: wild type.

## Competing interests

The authors declare that they have no competing interests.

## Authors' contributions

SY carried out the experiments and drafted the manuscript. VW carried out the immunoassays and participated in the animal studies. KM participated in the design of the study, the interpretation and helped to draft the manuscript. MTC conceived of the study, and participated in its design and coordination and helped to draft the manuscript. All authors read and approved the final manuscript.
